# Dietary Interventions in the Management of Fibromyalgia: A Systematic Review and Best-Evidence Synthesis

**DOI:** 10.3390/nu12092664

**Published:** 2020-08-31

**Authors:** Ethan Lowry, Joanne Marley, Joseph G. McVeigh, Emeir McSorley, Philip Allsopp, Daniel Kerr

**Affiliations:** 1 School of Health Sciences, Ulster University, Shore Road, Newtownabbey BT37 0QB, UK; lowry-e3@ulster.ac.uk (E.L.); j.marley@ulster.ac.uk (J.M.); 2 School of Clinical Therapies, University College Cork, Douglas Street, Cork T12 YN60, Ireland; joseph.mcveigh@ucc.ie; 3 School of Biomedical Sciences, Ulster University, Cromore Road, Coleraine BT52 1SA, UK; em.mcsorley@ulster.ac.uk (E.M.); pj.allsopp@ulster.ac.uk (P.A.)

**Keywords:** fibromyalgia, diet, nutrition, pain, sleep, rheumatology, musculoskeletal

## Abstract

Fibromyalgia syndrome (FMS) is characterised by chronic widespread pain alongside fatigue, poor sleep quality and numerous comorbidities. It is estimated to have a worldwide prevalence of 1.78%, with a predominance in females. Treatment interventions for fibromyalgia have limited success, leading to many patients seeking alternative forms of treatment, including modifications to their diet and lifestyle. The effectiveness of dietary changes in fibromyalgia has not been widely researched or evaluated. This systematic review identified twenty-two studies, including 18 randomised control trials (RCTs) and four cohort studies which were eligible for inclusion. In total these studies investigated 17 different nutritional interventions. Significant improvements in reported pain were observed for those following a vegan diet, as well as with the low fermentable oligo di-mono-saccharides and polyols (FODMAP) diets. Supplementation with *Chlorella* green algae, coenzyme Q10, acetyl-l-carnitine or a combination of vitamin C and E significantly improved measures of pain. Interpretation of these studies was limited due to the frequent poor quality of the study design, the wide heterogeneity between studies, the small sample size and a high degree of bias. Therefore, there is insufficient evidence to recommend any one particular nutritional intervention for the management of fibromyalgia and further research is needed.

## 1. Introduction

Fibromyalgia syndrome (FMS) is a condition characterised by chronic widespread pain, alongside fatigue, disturbed sleep and a combination of poor memory and poor concentration [1]. There is also a high prevalence of many co-morbidities [2], including: anxiety, depression [3], temporomandibular joint disorder (TMJ) [4], chronic fatigue syndrome (CFS) [5], migraines [6] and/or headaches [7], and irritable bowel syndrome (IBS) [8]. The exact combination of and severity of symptoms and comorbidities varies substantially between individuals. The aetiology and pathophysiology of FMS remains unclear; however, current literature supports the hypothesis of central sensitisation to be involved whereby pain signals and subsequent sensations are amplified within the pathways of the central nervous system (CNS). This often leads to many of the aforementioned symptoms and comorbidities found in FMS [9]. Regardless, fibromyalgia is a complex and multifaceted condition affecting each individual in a unique manner, with pain at the forefront. The experienced pain not only reduces overall quality of life but also has a significant impact on basic functions such as sleep and cognitive ability. This in turn exacerbates pain severity, leading to a “vicious-circle” of symptomatology and mental health problems, and a key feature of conditions characterised by central sensitisation [10,11].

There is no gold standard diagnostic process for fibromyalgia. Individuals are instead diagnosed with FMS if they meet the classification criteria drawn up by the American College of Rheumatology (ACR), first conceived in 1990 [12] and with three revisions; in 2010 [13], 2011 [14], and 2016 [15]: Prevalence, therefore, depends on which diagnostic criteria was used at the time [16]. Compared to the 1990 ACR criteria, the 2010 and 2016 revisions had a sensitivity of 86% and 89.5% respectively [15]. The most recent estimate of worldwide prevalence of FMS is 1.78%, with a mean prevalence of 3.98% in women and 0.01% in men [17]. FMS thus appears to be more prevalent in women, and, with age, those with low socioeconomic status, low education levels, and, those who live in rural areas [18].

There is also no gold standard treatment for FMS. Treatment regimens can and should vary significantly from person to person depending on their symptoms [19]. Despite this variability, individuals typically receive a combination of pharmacology, physiotherapy and cognitive behavioural therapy (CBT) [20]. Patients rarely experience full remission of symptoms, with only 25% noting any long-term improvements [21]. This may contribute to findings that individuals with FMS, routinely seek complimentary or alternative methods to control their symptoms [22]. Nutritional interventions or dietary changes are commonly used as alternative treatment approaches across many illnesses, including chronic pain conditions such as rheumatoid arthritis [23]. Despite a lack of research relating to the effect of dietary interventions or supplements, individuals with FMS frequently make dietary changes in an attempt to control their symptoms [24].

Dietary interventions may allow individuals with FMS to have an active role in the management of their condition and may fall within the scope of dieticians or nutritionists. Dietary intake as a whole, influences many physiological systems and processes, therefore, clinicians and patients alike should be aware of the dangers of following anecdotal evidence in regards to dietary interventions. Even if such changes positively influence fibromyalgia and its underlying mechanisms, it may negatively influence other physiology. Regardless, the ability for certain elements of nutrition to directly or indirectly affect the pathophysiology or symptoms of FMS, should not be ignored. There exist a number of potential mechanisms by which nutrition could be beneficial including but not limited to: oxidative status or damage; dysfunction of pro-inflammatory or anti-inflammatory modulation; dysfunction of energy production; or, dysfunction of the neuromodulation within the peripheral or central nervous systems. To recommend any nutritional intervention, will require extensive randomised, controlled, human trials. These will lead to informed and evidence-based choices and will therefor protect both the individual’s health and financial status. Currently there is a lack of conclusive data on any nutritional interventions. This paper aims to systematically review the existing literature and to explore dietary changes—including the use of nutritional supplements—as an intervention in the treatment of FMS as a whole and its many symptoms.

## 2. Methods

This systematic review was carried out in accordance with the Preferred Reporting Items for Systematic Reviews and Meta-Analysis guidelines checklist (PRISMA) [25]. A systematic search (Table 1) was carried out by one author (EL) using the electronic databases; Cinahl, MedLine, Scopus and Amed from 1990 until 20 March 2020. Papers were limited to journal articles and English-language papers. Title and abstracts were screened, removing any obvious papers. The remaining papers were read in full and the following eligibility/inclusion-exclusion criteria were then applied: Studies were excluded if they did not have an appropriate control group/measure. Adults, aged ≥18 years of age, if diagnosed with FMS by ACR criteria were included. Intravenous-nutrient therapies were excluded as such treatments are administered by medical professionals. Weight-loss diets were also excluded.

Papers suitable for inclusion were critically reviewed using the McMaster Critical Review Tool [26] as it allows the inclusion and comparison of multiple study designs, examining the following subsections: Study purpose, literature, design, sample, outcomes, intervention, results, and, conclusions and clinical implications. These subsections ask a total of 16 closed-ended questions, with the possible answers being: “yes”, “no”, “not addressed” or “n/a”. For a more objective comparison of study quality, the authors of this review have implemented the modified scoring system [27,28]. The scoring system allocates a score of “1” to the answer “yes” and a score of “0” to the answers “no”, “not addressed” and “n/a”. The maximum possible score was 16. Study scores were then categorised into the following: ≤8 = poor quality; 9–10 = fair quality; 11–12 = good quality; 13–14 = very good quality; and, 15–16 = excellent quality. Each paper was independently reviewed twice; once by the primary author (EL) then by a second reviewer (DK, PA, or EMcS). Differences were resolved by consensus.

## 3. Results

**Figure 1:**
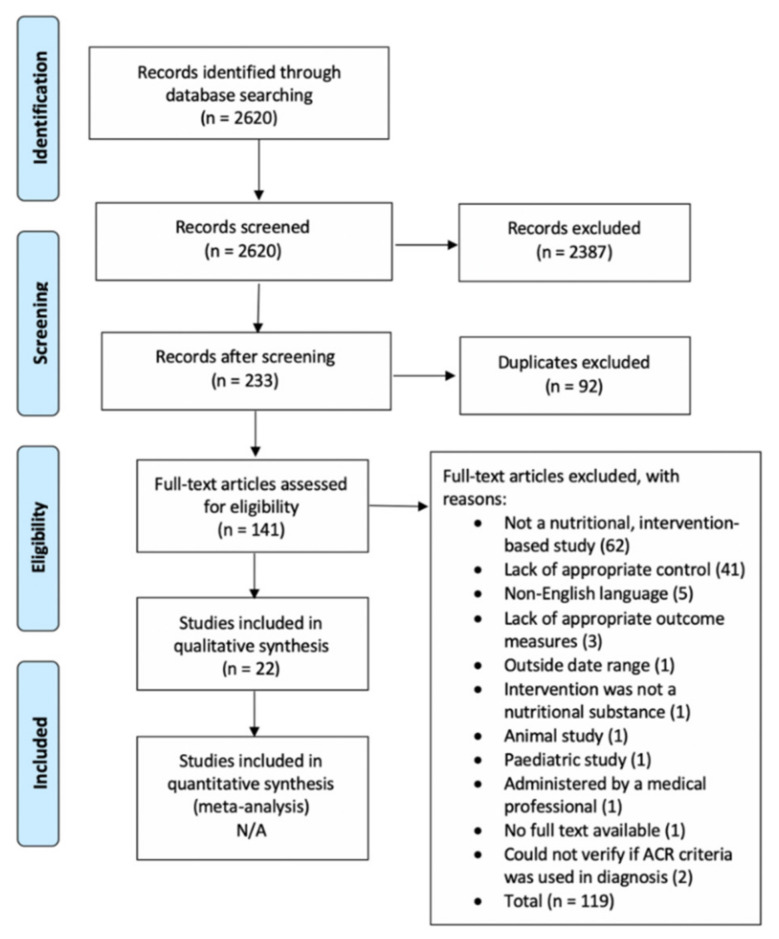
PRISMA 2009 Flow Diagram.

Initial search yielded a total of 2620 papers with 22 papers meeting all criteria. The results of the search are presented in a PRISMA flow diagram (Figure 1).

The 22 studies recruited 806 participants, including 17 males (2.06%) and 789 females (97.94%). Of the 22 studies, 15 (68%) exclusively recruited females. The mean number of participants was 37 (range 8–102). Illness duration within studies ranged from 4–11.8 years. The mean age range of participants was 39.6–58 years. Participant characteristics were recorded in Table 2. Seven studies investigated populations living in the United States of America [29,30,31,32,33,34,35]; five from Spain [36,37,38,39,40], two from Italy [41,42], and one from each of Portugal [43], Pakistan [44], Finland [45], Austria [46], France [47], Brazil [48], Turkey [49] and the United Kingdom [50].

Participants in 18 of the 22 studies were diagnosed using the 1990 ACR diagnostic criteria [10], one study used the 2010 ACR criteria [11], one used the modified 2010/2011 ACR criteria [12], and two used a combination of 1990 and 2010. In 16 of the 22 studies (73%) participants were both randomised and blinded. In total, blinding took place in 73% of studies, and randomisation took place in 82% of studies. With respect to study design, 18 RCTs were included, three of which involved crossover and four cohort studies, two of which involved crossover.

### 3.1. Intervention Characteristics

Across the 22 studies, there were 17 different dietary interventions (Table 3): coenzyme Q10 [38,39,41], vitamin D [33,46]; probiotics [40]; *Chlorella* green algae [35]; vegan diet [45]; tart cherry juice [30]; low-FODMAP diet [43]; soy [34]; extra-virgin olive oil [37]; caffeine [32]; elimination of MSG [31]; elimination of MSG and aspartame [36]; vitamin C, E & *nigella sativa* seeds [44], vitamins C and E [49], creatine [48] and acetyl-l-carnitine [42]. Finally, two studies investigated phytotherapy-based supplements which included a myriad of different plant extracts [47,50] and one study investigated a phytotherapy-based supplement alongside an elimination diet [29]. A range of controls were implemented: placebo control, standard diet, waiting list control, healthy controls to compare against participants bloodwork, and conventional treatment.

### 3.2. Study Quality

Study quality as measured using the McMaster Critical Review Tool for Quantitative Studies, ranged from a score of 8/16 (poor study quality) [29] to 14/16 (very good study quality) [48]. The average score across the studies was 11/16 (good quality). One paper was found to have “poor” study quality [29]. Six papers were found to have “fair” study quality [36,41,43,44,49,50]. Eight papers were found to have “good” study quality [30,31,37,38,39,42,45,46]. Seven papers were found to have “very good” study quality [32,33,34,35,40,47,48].

Only four papers presented as having no obvious bias [30,32,34,46]. Six reportedly performed sample size calculations [33,35,40,42,47,48]. No study reported the reliability of their outcome measures; however, six papers did report on the validity of their outcome measures [33,34,39,43,48]. A total of five papers explicitly reported their findings in terms of clinical importance [32,37,38,39,48].

### 3.3. Outcome Measures

Across the 22 studies there was a total of 80 different outcome measures. These outcome measures can be categorised into the following areas: disease severity, general health, pain, mental health; sleep, fatigue and tiredness; strength, stiffness and exercise tolerance; gastrointestinal symptoms or conditions, cognitive function, and miscellaneous. The three most common outcome measures were, in descending order: VAS-Pain (63.64%), Fibromyalgia Impact Questionnaire (50%) and Tender Point Evaluation (36.36%). The following results have been reported in terms of statistical and clinical significance using *p* values, and, if published, specific values for the outcome measure being discussed. Otherwise, percentage changes were reported.

### 3.4. Pain

A total of 20 of the 22 studies (91%) specifically evaluated the symptom of pain using one or more of 15 different outcome measures. Significant improvements were noted in VAS-Pain after the consumption of *Chlorella* green algae (reduction of 31%; *p* < 0.001) [35], a low-FODMAP diet (6.6/10 to 4.9/10; *p* < 0.01) [43], consumption of coenzyme Q10 (reduction of 56% *p* < 0.01) [39], or, the consumption of a supplement regimen which included vitamin C, E and *Nigella sativa* seeds (90.3 ± 1.52 to 77.80 ± 1.65; *p* < 0.05) [44]. Supplementation of acetyl-l-carnitine (*p* < 0.001) [42] and consumption of a vegan diet (*p* = 0.005) [45] also resulted in significant improvements in VAS-Pain but neither the exact scores or percentage changes in scores were published. Supplementation of vitamin D [46] lead to a consistent decrease in VAS-Pain compared to relatively constant scores with the placebo group (68.70 ± 12.53 to 53.40 ± 29.31 vs. 62 ± 20.28 to 64.50 ± 16.14) as measured via two (groups) four (time points) variance analysis (*p* = 0.025). However, after supplementation ceased, no significant difference was noted between groups. The minimum clinically important differences (MCID) for VAS-Pain is a reduction of 1.1 points on an 11-point scale, or, an 11-point reduction on a 100-point scale [51]. Significant reduction in the number of tender points was recorded after the consumption of *Chlorella* green algae [35] (15.5 ± 2.3 to 14.4 ± 2.8; *p* = 0.009), supplementation of coenzyme Q10 [39] (reduction of 44%; *p* < 0.01). Supplementation of acetyl-l-carnitine [42] also resulted in a significant improvement in Total Myalgic Score (*p* < 0.05) and mean number of tender points (*p* < 0.05) but again no specific scores were published.

### 3.5. Fibromyalgia Severity

A total of 13 of the 22 studies (51%) measured overall fibromyalgia severity. Fibromyalgia Impact Questionnaire (FIQ) scores were significantly improved following the consumption of extra-virgin olive oil vs. refined olive oil [37] (68.61 ± 7.17 to 52.47 ± 9.68 vs. 47.84 ± 7.47 to 46.83 ± 4.87; *p* < 0.001). Supplementation of coenzyme Q10 [39] resulted in a significant improvement of FIQ scores (52% reduction; *p* < 0.001). The Revised Fibromyalgia Impact Questionnaire (FIQR) scores were significantly worsened through the reintroduction of monosodium glutamate versus placebo [31] (22.20 ± 20.60 to 48 ± 22.40 vs. 22.20 ± 20.60 to 35.70 ± 19.40; *p* < 0.03). The MCID of the FIQ is a 14% change (14 points) in total score [52]. Fibromyalgia Severity Questionnaire (FSQ) scores were significantly improved following the implementation of a low-FODMAP diet [43] (21.8 to 16.9; *p* < 0.01). FSQ scores, however, did not significantly worsen following the reintroduction of FODMAPs.

### 3.6. General Health

General health outcome measures were recorded in nine of the 22 studies. A statistically significant treatment effect was noted in the General Health Questionnaire (GHQ28) in response to three separate doses (120 80 and 40 mg per day) of phytonutrient rich supplements [50]. Only a dosage of 80 mg was significantly different compared to placebo (6.78 ± 7.59 to 1.56 + 2.40 vs. 6.78 ± 7.59 to 6.56 ± 6.71). In a two-arm, crossover study supplementation of coenzyme Q10 versus placebo, the physical health index and mental health index of the SF-36 were significantly improved (*p* < 0.05), nevertheless this was only observed when coenzyme Q10 was given first, but not when coenzyme Q10 was given after crossover. Of the eight SF-36 subscales, only “physical pain” resulted in significant improvements (*p* < 0.05) after the consumption of coenzyme Q10 in both arms [41]. Consumption of a vegan diet [45] lead to significant improvements in General Health Questionnaire (GHQ) (*p* = 0.02) and Health Assessment Questionnaire (HAQ) (*p* = 0.03) however no specific scores were published. Compared to placebo, supplementation with acetyl-l-carnitine [42] lead to significant between-group improvements of three of the eight SF-36 subscales: bodily pain, mental health and general health perception; and, both the physical and mental health total scores of the SF-36. No specific scores, however, were published, with the authors visually displaying changes through figures. Compared to placebo, supplementation of either 1200 IUs or 2400 IUs of vitamin D [46] resulted in the significant improvement of the physical role functioning subscale of the SF-36 (*p* = 0.022) but no significant improvements for any of the remaining seven subscales, nor the mental health or physical health totals. No specific SF-36 scores were published. The MCID for the SF-36 mental and physical health indexes (MCS and PCS) in FMS patients is a change of six points, indicating a substantial positive change in both physical and mental health [53].

### 3.7. Mental Health

Nine of the 22 studies investigated outcome measures related to mental health and/or depression and anxiety. Consumption of *Chlorella* green algae [35] versus placebo resulted in significant improvements in the Hassles anxiety scale (50.2 ± 38.5 to 37.2 ± 36.9 vs. 50.3 ± 43.3 to 42.9 ± 38.3; *p* = 0.01). Consumption of a phytonutrient rich supplement [47] resulted in a significant improvement (mean difference −8.06; *p* < 0.001) in SF-12 Mental Health Summary Scale scores when compared to a comparative food supplement (*p* = 0.02) and with no supplementation at all (*p* = 0.018). Significant improvements were also recorded in Hospital Anxiety and Depression scores (mean difference −2.00; *p* = 0.004) when compared to a comparative food supplement (*p* = 0.013) and with no supplementation at all (*p* = 0.007). Supplementation of coenzyme Q10 [38] lead to a significant improvement in Becks Depression Inventory scores vs. placebo (post intervention scores 6.2 ± 1.9 vs. 24.1 ± 3.5; *p* < 0.001) however, no baseline scores were published. Consumption of a low-FODMAP diet [43] resulted in a significant reduction in VAS-Depression scores (5.1 to 4.2; *p* < 0.05). Supplementation of acetyl-l-carnitine [42] also lead to a significant improvement in VAS-Depression (*p* < 0.05) nevertheless specific scores were not published. Consumption of extra-virgin olive oil resulted in a significant improvement in the MCS-12 when compared to refined olive oil (31.62 ± 2.35 to 36.20 ± 2.94 vs. 49.10 ± 3.42; *p* = 0.017; *p*.Adj = 0.035) [37]. The mental health subscale of the SF-36 was significantly improved after supplementing with creatine compared to placebo [48] (+23%; *p* = 0.03).

### 3.8. Sleep, Fatigue & Tiredness

Eight of the 22 studies recorded sleep, fatigue or tiredness outcomes. Consumption of *Chlorella* green algae [35] resulted in significant improvements of the general patient questionnaire (PAQ) VAS subscales for sleep (18% improvement; *p* = 0.047) and fatigue (20% improvement; *p* = 0.005). Pittsburgh Sleep Quality Index (PSQI) (mean difference −3.00; *p* = 0.002) and Pichot fatigue scores (mean difference −2.00; *p* = 0.004) were significantly improved in response to phytonutrient supplementation [47]. Three separate doses (120 mg/80 mg/40 mg) of a phytonutrient supplement [50] resulted in significant improvements in a Likert scale for sleep quality when compared to placebo (Mean difference −0.27 *p* = 0.04; −0.40 *p* = 0.004; and, −0.32 *p* = 0.02): but no significant changes in levels of fatigue when reported by the patient. When recorded by the investigator a dosage of 80 mg resulted in a significant improvement in fatigue (2.56 ± 0.88 to 1.78 ± 0.67; *p =* 0.01) but no significant improvements in sleep. VAS-Sleep Quality was significantly improved (6.6 to 5.1; *p* = 0.017) after the consumption of a low-FODMAP diet [43]. Investigation of a vegan diet [45] resulted in significant improvements (*p* = 0.0001) in a non-validated sleep questionnaire, however no specific scores were published, nor were the exact questions used in the questionnaire.

### 3.9. Strength, Stiffness & Exercise Tolerance

Six of the 22 studies documented the effects on strength, stiffness or exercise tolerance. Implementation of a vegan diet [45] lead to a positive significant (*p* = 0.001) change in a stiffness “questionnaire” however, no specific scores were given and the exact outcome measure used was not disclosed. Creatine supplementation [48] versus placebo lead to a significant increase in muscle strength during leg press (+9.8% vs. −0.5%; *p* = 0.02) and chest press (+1.2% vs. 7.2%; *p* = 0.002) exercises; and isometric strength (+6.4% vs. −3.2%; *p* = 0.007). Aerobic exercise was not significantly changed.

### 3.10. Gastrointestinal Symptoms or Comorbidities

Three studies recorded the effects on gastrointestinal symptoms. Implementation of a low-FODMAP diet [43], lead to a significant improvement in IBS Severity Score (275.3 to 137.4; *p* < 0.01). Elimination and subsequent reintroduction of monosodium glutamate [31] versus placebo resulted in significantly improved IBS-Quality-of-life scores (25.5 ± 20.4 vs. 17.5 ± 14.7; *p* < 0.05) but did not result in a significant improvement of VAS-IBS.

### 3.11. Cognitive Function

Three of the 22 studies investigated cognitive function. Implementation Low-FODMAP diet [43] resulted in significantly improved scores for VAS-Memory (6.9 to 5.0; *p* = 0.001). Probiotic supplementation [40] resulted in significant improvements in cognitive function through the Two-Choice Task, indicating a reduction in the number of impulsive choices as measured by repeated measures ANOVA, Group by Time interaction (*p* = 0.029). 

## 4. Discussion

This review of 22 nutritional intervention studies provided conflicting results across a multitude of outcome measures. Pain which is often regarded as the characterising symptom of FMS was significantly improved after the consumption of: *Chlorella* green algae, vegan diet, coenzyme Q10, acetyl-l-carnitine; a low-FODMAP diet; and, a combination of vitamin C, E and *Nigella sativa* seeds.

The baseline characteristics of participants recruited within the studies are in keeping with other fibromyalgia intervention studies and in keeping with the typical characteristics in the wider population of individuals with fibromyalgia [54]. Participant numbers were generally quite low (mean = 37). Sample sizes of the studies included in this review ranged from eight to 75, however, only six of the 22 studies justified their sample size [33,35,40,42,47,48] based on power calculations or previously published fibromyalgia intervention studies therefor, the significant findings found among many of the included studies should therefore be interpreted with caution [55]. Further research in nutritional intervention studies should not only seek to recruit larger samples, but also ensure it is statistically sound in terms of being sufficiently powered. Otherwise, the ‘significant’ findings as found within this review, may not be significant at all when applied to the wider demographic.

Dosage and duration of any intervention can have a significant impact on their efficacy. Due to the heterogeneity of the specific interventions across this review, there was some variability in terms of the exact dosage and duration, even within the same intervention. The use of nutritional interventions in alleviating symptoms that the nutrient is not primarily associated with contributes to the difficulty in analysing efficacy. For example, the pathophysiology of how vitamin D regulates calcium absorption is very well understood, which allows researchers to carefully look at specific biomarkers and dosage, in determining the effect that dietary intake of vitamin D may have. Conversely, the pathophysiological understanding of how vitamin D may affect pain is poorly understood, thus determining dosage and duration of intervention studies for pain is much more difficult. A common approach is to refer to recommended intakes and/or nutritional status. In some instances, however, it may not always be possible to target a specific measure of nutritional status for the corresponding intervention, such as the vegan diet included within this review [45]. A dietary protocol such as that described simply involves the exclusion of animal products and there were no restrictions on the ‘dosages’ of the food(s) the participants were permitted to consume. The recording of the nutritional status of the specific nutrients being investigated is of paramount importance when analysing the effect of nutritional interventions [56]. Researchers need to demonstrate if their chosen demographic present with deficiencies in the nutrient being investigated and ensure that their chosen dosage results in, first and foremost a meaningful change in nutritional status, and, that this change is maintained until the subjects nutritional status meets the recommended concentrations. Unfortunately, these methods are not commonly implemented, again suggesting caution when interpreting results. Interventions such as soy [34], olive oil [37], tart cherry juice [45] or the phytonutrient regimens [29,35,47,50] included within this review contain many nutrients and fall short of being able to accurately measure any one particular nutrient to ascertain pre and post nutritional status. Finally, several of the studies included within this current review involved elimination diets [31,36,43], eliminating FODMAPs, glutamate and aspartame; of which none documented pre and post nutritional status of these substances.

There was evidence of selection/recruitment bias across several of the included studies with others having no information at all with regards to the recruitment/sampling process. Seven studies recruited directly from active treatment groups within their geographical area [34,41,42,43,44,46,49].

One of the biggest limitations of this review was the heterogeneity among studies, especially with regards to outcome measures. Fibromyalgia as a condition incorporates a myriad of symptoms and comorbidities which can vary significantly among sufferers. In particular, individuals with fibromyalgia have been known to report significant variability in pain, mood and fatigue [57]. The outcome measures used to record pain levels varied significantly with the Visual Analogue Scale (VAS) for example was used in 63.64% of the included studies. The main benefit of the VAS is the speed of which it can be implemented however it has a key limitation in that it requires specific context when put towards a participant e.g., current pain level, pain level in the last 24 h, low back pain or headache pain. As pain manifests itself in many different ways, one could argue that a multidimensional scale such as the McGill Pain Questionnaire [58] may be more appropriate.

Unless investigating specific nutrients or ingredients, blinding of participants in dietary or nutritional intervention studies, using sufficient control measures is challenging. This is made more difficult if the intervention involves an overarching dietary principle such as those in this systematic review: vegan diet [45], low-FODMAP diet [43], or elimination diets [29,31,36]. The majority of studies which *did* facilitate blinding or randomisation of participants, lacked specific details as to how this was achieved. Volunteer bias existed in several studies; in particular, the individuals who chose the vegan dietary intervention being studied, had significantly higher levels of pain [45]. This may indicate that those with more severe symptoms are more likely to seek complementary or alternative forms of treatment. 

The theory of treating a condition like fibromyalgia using nutritional interventions is complex. There is a lack of understanding as to how the intervention may affect the underlying pathophysiology. Understanding of the mechanisms involved is made more difficult as at least some symptoms and comorbidities appear to directly influence each other and thus may lead to unnecessary or frivolous attempts at treating the condition [59]. Additionally, having fibromyalgia does not exclude an individual from also developing other conditions; making it difficult for patients and clinicians to differentiate what can be attributed to fibromyalgia, thus providing further confusion in regards to treatment. For example, an individual who has fibromyalgia, may also receive a diagnosis of osteoarthritis (OA) and/or rheumatoid arthritis (RA). There is some evidence that n-3 long chain polyunsaturated fatty acids (LCPUFA) may reduce pain in those diagnosed with both OA and RA [60,61,62]. Currently it would be difficult for a sufferer of both these conditions to differentiate between these, and fibromyalgia; therefore attributing the treatment effect of a nutritional intervention would be equally as difficult.

The majority of studies included within this review lack a full understanding as to how their proposed nutritional intervention may influence pathophysiology. Despite this, there are several hypothetical pathways in which the interventions reviewed may influence the pathophysiology and/or symptomology of fibromyalgia. These include: amelioration of oxidative stress, reduction of inflammatory markers; amelioration of mitochondrial dysfunction and, removal of dietary “toxins.” Several interventions cross over a number of these. Table 4 identifies the interventions included within this review and their hypothesised mechanisms and physiological benefit. When results were analysed in terms of each of these hypothesised mechanisms, no one mechanism appeared to be any more or any less promising. Without investigating corresponding biomarkers for each of these mechanisms it would be speculative to suggest any one mechanism supersedes the rest.

Individuals with FMS have been found to have significantly higher levels lipid perioxidation in their skin [63] and significantly higher total oxidant status and serum prolidase in fasting blood samples [64] which are also positively correlated with VAS-Pain and VAS-Fatigue. Oxidative stress also plays a pathophysiological role within the neuropathic pain of diabetic neuropathy [65] and chemotherapy-induced neuropathy [66]. There is also evidence that oxidative stress correlates significantly with specific inflammatory biomarkers such as Tumour Necrosis Factor Alpha (TNF-α) and C-Reactive Protein (CRP) in individuals with rheumatoid arthritis [67].

Significant positive correlations were also noted with markers of oxidative stress and inflammatory cytokine interleukin (IL)-6 in patients with Major Depressive Disorder [68]. CRP and TNF-α have also been found to be significantly higher in individuals with fibromyalgia compared to healthy controls [69,70] and have been found to correlate positively with pain and fatigue [71]. The relationship between oxidative stress and inflammatory changes is complex with both mechanisms being interdependent. A pathology manifesting as a primary oxidative stress condition will eventually lead to inflammation which further exacerbates levels of oxidative stress and vice versa [72]. For optimum outcomes it is proposed that chronic patients may benefit from the treatment of both.

More specifically, oxidative stress has also been shown to exacerbate *neuroinflammatory* changes as evidenced in neurodegenerative disorders [73] and may have a similar effect within fibromyalgia. Evidence does exist of systemic inflammation, and specifically, central neuroinflammation in individuals with fibromyalgia [74]. Animal models have shown that oxidative stress leads to the activation of spinal microglial cells leading to the overexpression of TNF-α, IL-1β and IL-6 [75]. Furthermore, brain glial activation has been noted in both fMRI and PET imaging in individuals with fibromyalgia [76]. This may in part account for the aforementioned central neuroinflammation in humans. Such inflammatory changes could hypothetically lead to increased phosphorylation of N-methyl-D-aspartic receptors in spinal dorsal horns thus playing a role in the central sensitization of fibromyalgia [75]. Diets high in consumption of fruit and vegetables have been shown to reduce oxidative stress [77]. Furthermore, dietary interventions such as soy protein, n-3 LCPUFA, low-fat-low-carbohydrate diet and grape polyphenols have been successful in reducing inflammatory biomarkers including TNF-α and CRP [78]. Consumption of a Mediterranean diet resulted in significantly lower IL-6 and CRP concentrations individuals with metabolic syndrome when compared to controls [79]. Individuals who consume a diet high in fruits, vegetables, whole grains, white meat, tomato, legumes, tea and fruit juices were significantly and inversely related to markers of systemic inflammation even after controlling for BMI and waist circumference. Conversely, diets rich in refined grains, red meat, butter, processed meat, high-fat dairy, sweets, desserts, pizza, potatoes, eggs, hydrogenated fats and soft drinks were found to be significantly and positively correlated to markers of systemic inflammation [80]. This may account for the positive changes noted within this review from the vegan diet [45], chlorella green algae [35] or extra-virgin olive oil [37].

Individuals with FMS have also been shown to present with mitochondrial dysfunction [63,81] and significantly reduced coenzyme Q10 concentrations [82,83]. Further evidence suggests that levels of oxidative stress and mitochondrial dysfunction, through coenzyme Q10 levels are correlated with fibromyalgia symptoms [84]. Supplementation of coenzyme Q10 has been shown within this review to improve these markers and the symptoms of FMS [38,39,41]. Animal models [85] have demonstrated a significant improvement in oxidative status and inflammatory markers after the consumption of acetyl-l-carnitine. L-Carnitine is routinely converted to acetyl-l-carnitine and back again depending on the cells’ requirements. Supplementation of L-carnitine has demonstrated significant improvements in oxidative status of human participants [86]. Future nutritional intervention studies should account for the lack of physiological understanding and facilitate ways in which to measure the appropriate biomarkers to either support or negate their hypothesis. 

This review concludes that there is insufficient evidence to recommend any one particular dietary intervention in the management of FMS symptoms. Most studies lack power calculations and have a small number of participants. Widespread heterogeneity exists across study methodology, particularly with regards to intervention protocol and outcome measures making comparisons between studies very difficult. Study quality was relatively good across the included studies; however, the majority had a high risk of bias, with several also lacking blinding and/or randomisation. Finally, several papers did not publish specific values for outcome measures making comparisons with other interventions difficult. Because of these limitations, the statistically significant findings should be interpreted with caution, especially in terms of their clinical significance. These findings are in keeping with a recent systematic review by Silva and colleagues [87].

Conventional therapies in the treatment of fibromyalgia. Despite these substantial limitations, a reduction in pain and overall severity of fibromyalgia was observed in those studies who supplemented with *Chlorella* green algae, coenzyme Q10, acetyl-l-carnitine, a vegan diet, extra-virgin olive oil, a low-FODMAP diet, a combination of vitamin C, E and *Nigella sativa* seeds. Each of these interventions have been shown to be involved in improving oxidative status, energy production and inflammatory markers. Coenzyme Q10, in particular, appears promising as it has been associated with benefits to oxidative stress, energy metabolism and in regulating inflammation. Further research is required across each of the interventions within this review, with statistically sound sampling methodologies and measurements of oxidative stress and inflammatory biomarkers to further bolster pathophysiological understanding. Further research may also benefit from investigating certain combinations of nutritional modalities. For example, a nutritional intervention grounded in an anti-oxidative mechanism combined with one with an anti-inflammatory mechanism, versus anti-oxidative or anti-inflammatory alone. This may demonstrate the interplay within and between such systems and how this may or may not affect the pathophysiology of fibromyalgia. If these interventions show further promise in well-designed clinical trials if may provide an active treatment protocol to use with or without.

## Figures and Tables

**Table 1:** Search strategy.

Search Number	Search Terms
Search #1	“Fibromyalgia” OR “FMS” OR “Fibrositis”
Search #2	“Diet therapy” OR “Diet” OR “Nutrition” OR “Vitamin(s)” OR “Minerals” OR “Micronutrients” OR “Macronutrients” OR “Dietary Supplement” OR “Dietary Supplementation” OR “Food and Beverages” OR “Vegetarian” OR “Vegan” OR “Dietary Fats” OR “Dietary Carbohydrates” OR “Dietary Proteins” OR “Coenzyme(s)”
Search #3	Search #1 AND Search #2

**Table 2:** Participant Baseline Characteristics.

Paper	Intervention	ACR Criteria	Sample (n)		Age (Years), Mean (SD)		% Female	
			Tr	C	Tr	C	Tr	C
[45]	Vegan diet *	1990	18	15	51	52	100	100
[30]	Tart cherry juice *	1990	8	7	51*		100	100
[43]	Low-FODMAP diet	2011	38		51*		100	
[29]	Phytonutrient supplement	1990	8		55.6 (9.4)		100	
[34]	Soy	1990	25	25	Median ^+,^*, 47.7		98	
[37]	Extra-Virgin olive oil	1990	11	12	53.63 (5.50)	48.16 (7.96)	100	100
[36]	Elimination of MSG and aspartame	1990	36	36	42.33 (8.43)	39.64 (8.16)	100	100
[38]	Coenzyme Q10	1990	10	10	--	--	--	--
[33]	Vitamin D	1990	20	22	58.0 (7.3)	56.7 (11.3)	98	87
[44]	Vitamin C, E & *Nigella* seeds	1990	50		42.93 (1.59)		100	
[32]	Caffeine	1990	23		43.57 (18.49)		86.96	
[46]	Vitamin D	1990 + 2010	15	15	48.37 (5.301)		90	
[41]	Coenzyme Q10	2010	12	10	52.5 (10.4)	53.6 (7.8)	100	100
[48]	Creatine	1990	15	13	48.7 (8.4)	49.0 (10.1)	100	100
[31]	Elimination of MSG	1990	31		53.4 (13)		90	
[39]	Coenzyme Q10	1990	10	10	44.3 (9.7)	55 (5)	100	100
[42]	Acetyl-l-carnitine	1990	50	52	47.3 (11.7)	46.3 (10.4)	97	
[50]	Phytonutrient supplement	1990	12		45.6 (5.9)		100	
[49]	Vitamin C and E	1990	31	30	40.1 (5.2)	39.6 (5.8)	100	100
[35]	Chlorella green algae	1990	30		47.1 (9.0)		97	
[40]	* Phytonutrient supplement	1990 + 2010	31		55	50.27	94	87
[47]	Probiotics	1990	75 (3-Arm)		A: 49.6 (9.4) B: 47.4 (8.6)	47.8 (9.0)	100	100

Legend: Tr = Treatment group(s); C = Control group; A = Treatment group “A”; B = Treatment group “B”; N = Number; SD = Standard Deviation; * = No Standard deviation given; + = No mean given; MSG = monosodium glutamate; FODMAP = fermentable oligo di-mono-saccharides and polyols.

**Table 3:** Study Intervention and statistically significant results.

Paper	Intervention (Dosage)	Duration	Statistically significant results (*p* < 0.05)
[45]	Vegan diet compared to participants normal diet	3 months	Improved VAS-Pain; morning stiffness; GHQ; HAQ; & Sleep quality
[30]	Tart cherry juice (2 x 10.5 Oz bottles daily) or placebo	2 weeks	No statistically significant changes
[43]	A diet low in Fermentable oligo-di-mono-saccharides and polyols (Low-FODMAP). Reducing consumption of lactose, excess fructose, fructans, galactans, polyols.	4 weeks	Improved: VAS-pain; VAS-muscle tension; VAS-asthenia; VAS-depression; VAS-sleep quality; VAS-memory; VAS-headache; VAS-abdominal pain; VAS-constipation; VAS-diarrhoea; VAS-Bloating; FSQ; and, FIQR; IBS-SSS
[29]	Phytonutrient supplement containing: 3 g fat, 20 g carbs, 6 g sugars, 12 g protein; 4000 IU β-carotene; 1000 IU vitamin A; 300 mg vitamin C; 35 IU vitamin D; 42 IU vitamin E; 2 mg thiamine; 2 mg riboflavin; 7 mg niacin; 3.4 mg vitamin B6; 80 μg folate; 2.6 μg vitamin b12; 135 mg biotin; 36 mg pantothenic; 220 mg sodium; 520 mg potassium; 1 mg iron; 230 mg phosphorus; 53 μg iodine; 160 mg magnesium; 10 mg zinc; 1 mg copper; 1 mg manganese; 50 μg chromium; 20 mg sulfate; 1 g spent hops; 50 mg pomegranate rind extract 125 mg prune skin extract; 67 mg watercress whole plant extract; 15 mg decaffeinated green tea extract (2 x daily servings) Elimination of: simple sugars, artificial colours, flavours and sweeteners; caffeinated beverages; gluten; eggs, dairy; allergenic foods; or foods high in arachidonic acid	4 weeks	Improved: FIQ subsections for pain and stiffness
[34]	Soy protein (20 g), soy isoflavone (160 mg) (1 serving daily) or placebo	6 weeks	Both soy and placebo resulted in significant improvements in FIQ and CES-D. No significant differences between groups
[37]	Extra-virgin olive oil (50 mLs) vs. refined olive oil	2 weeks	Improved: MCS-12 and FIQ
[36]	Elimination of MSG and aspartame from diet	3 months	No statistically significant changes
[38]	Coenzyme Q10 (300 mg daily) or placebo	40 days	Improved: BDI
[33]	Vitamin D (50,000 IU once per week) or placebo	3 months	No significant changes compared to placebo
[44]	Vitamin C (200 mg daily), E (200 mg daily) & *Nigella sativa* seeds (13 mg 4–5 times daily)	8 weeks	Improved: VAS-pain
[32]	Caffeinated chewing gum (100 mg caffeine) or placebo	1 x serving	No significant changes compared to placebo
[46]	Vitamin D (1200 IU or 2400 IU daily) or placebo	25 weeks	Improved: VAS-Pain and FIQ subsection for morning fatigue
[41]	Coenzyme Q10 (400 mg daily) or placebo	6 months	Improved: SF-36 Subscale for physical pain
[48]	Creatine (20 g daily for 5 days; followed by 5 g daily) or placebo	16 weeks	Increased: muscle strength leg press and chest press; and isometric strength.
[31]	Elimination of MSG from diet	3 days	Worsened: symptom frequency; IBS-QOL; FIQR after the consumption of MSG
[39]	Coenzyme Q10 (300 mg daily) or placebo	40 days	Improved: FIQ; VAS-pain; TPE
[42]	Acetyl-L-carnitine (2 x 500 mg capsules daily and 1 x 500 mg IM injection weekly for 2 weeks; 3 x 500 mg capsules daily for 8 weeks) or placebo	10 weeks	Improved: TPE; Total myalgic score; VAS-pain, VAS-depression; SF-36
[50]	Colladeen™ Anthocyanidin Phytonutrient supplement: grape seeds, bilberries and cranberries. (120 mg a day/80 mg a day/40 mg a day/placebo) 12 weeks per dosage + 4 weeks baseline period	52 weeks	Improved: Likert scale-sleep; GHQ-28
[49]	Vitamin C (500 mg) & E (150 mg daily)	12 weeks	No significant changes
[35]	*Sun Chlorella*™ green algae tablets (10 g x 50 daily) and Wakasa Gold Chlorella™ (100 mL daily) or placebo	3 months	Improved: PAQ; VAS-Pain; TPE; Hassles scale
[40]	Ergyphilus Plus™ Probiotics: *Lactobacillus rhamnosus* GG, *Casei, Acidophilus* and Bifidobacterium *Bifidus* (2 pills with breakfast and dinner) or placebo	8 weeks	Reduced: number of impulsive choices (within the “two-choice task”)
[47]	Phytonutrient supplement (Fib-19-01) morning pill: ginger extract 50 mg, acerola 240 mg, vitamin C 120 mg, meadowsweet 40 mg, royal jelly 40 mg (one capsule). Phytonutrient supplement (Fib-19-01) evening pill: passiflora 80 mg, camomile 80 mg, meadowsweet 40 mg, quackgrass 100 mg and L-tyrosine 45 mg (1 capsule). Food supplement comparator: magnesium 71 mg, valerian 65 mg, escholtzia 50 mg, white ginseng roots 83 mg, willow 50 mg, acerola 120 mg, sage 50 mg and L-tryptophan 220 mg (1 capsule in morning and 1 capsule in evening) or no supplementation at all.	24 weeks	Improved: Pichot scale; HAD; SF-12 Subsections for Mental and social score variations when compared to food supplement comparator and no supplementation Improved: FIQ for Fib-19-01 but not significant when inter-group comparison took place

Legend: GHQ = General Health Questionnaire; VAS = Visual Analogue Scale; FSQ = Fibromyalgia Survey Questionnaire; FIQ = Fibromyalgia Impact Questionnaire; FIQR = Revised Fibromyalgia Impact Questionnaire; IBS-SSS = Irritable Bowel Syndrome—Symptom Severity Scale; CES-D = Centre for Epidemiological Studies for Depression Scale; MCS-12 = SF-12 Subscale—Mental Health Component Score; FACIT = Functional Assessment of Chronic Illness Therapy; HAQ = Health Assessment Questionnaire; PSQI = Pittsburgh Sleep Quality Index; GHQ-28 = General Health Questionnaire; PAQ = General Patient Questionnaire; TPE = Tender Point Evaluation; HAD = Hospital Anxiety and Depression Scale; IBS-QOL = Irritable Bowel Syndrome—Quality of Life; BDI = Becks Depression Inventory.

**Table 4:** Hypothesised physiological mechanisms.

Intervention	Mechanism			
	Antioxidant	Anti-Inflammatory	Energy Production	Immuno-Neuromodultion
Phytotherapy	+			
Probiotic		+		+
*Chlorella* green algae	+			+
Vegan diet	+	+		
Tart cherry juice	+	+		
Low-FODMAP	+			+
Soy		+		+
Extra-virgin olive oil	+	+		
Vitamin D		+		+
Caffeine				+
Vitamin C, E and *Nigella sativa*	+			
Vitamin C and E	+			
Creatine			+	
Coenzyme Q10	+	+	+	
Acetyl-L-carnitine	+	+		
Elimination of MSG and aspartame				+
Elimination of MSG				+

Legend: MSG = Monosodium Glutamate.
